# Multi-material three dimensional printed models for simulation of bronchoscopy

**DOI:** 10.1186/s12909-019-1677-9

**Published:** 2019-06-27

**Authors:** Brian Han Khai Ho, Cecilia Jiayu Chen, Gerald Jit Shen Tan, Wai Yee Yeong, Heang Kuan Joel Tan, Albert Yick Hou Lim, Michael Alan Ferenczi, Sreenivasulu Reddy Mogali

**Affiliations:** 1 Lee Kong Chian School of Medicine, Nanyang Technological University, 11 Mandalay Road, Singapore, 308232 Singapore; 2 Diagnostic Radiology, Tan Tock Seng Hospital, Singapore, Singapore; 3 Singapore Centre for 3D Printing, School of Mechanical and Aerospace Engineering, Nanyang Technological University, Singapore, Singapore; 4 Respiratory and Critical Care Medicine Clinic, Tan Tock Seng Hospital, Singapore, Singapore

**Keywords:** 3D printing, simulation, bronchoscopy, bronchial tree, multi-material, airway pathology

## Abstract

**Background:**

Bronchoscopy involves exploration of a three-dimensional (3D) bronchial tree environment using just two-dimensional (2D) images, visual cues and haptic feedback. Sound knowledge and understanding of tracheobronchial anatomy as well as ample training experience is mandatory for technical mastery. Although simulated modalities facilitate safe training for inexperienced operators, current commercial training models are expensive or deficient in anatomical accuracy, clinical fidelity and patient representation. The advent of Three-dimensional (3D) printing technology may resolve the current limitations with commercial simulators. The purpose of this report is to develop and test the novel multi-material three-dimensional (3D) printed airway models for bronchoscopy simulation.

**Methods:**

Using material jetting 3D printing and polymer amalgamation, human airway models were created from anonymized human thoracic computed tomography images from three patients: one normal, a second with a tumour obstructing the right main bronchus and third with a goitre causing external tracheal compression. We validated their efficacy as airway trainers by expert bronchoscopists. Recruited study participants performed bronchoscopy on the 3D printed airway models and then completed a standardized evaluation questionnaire.

**Results:**

The models are flexible, life size, anatomically accurate and patient specific. Five expert respiratory physicians participated in validation of the airway models. All the participants agreed that the models were suitable for training bronchoscopic anatomy and access. Participants suggested further refinement of colour and texture of the internal surface of the airways. Most respondents felt that the models are suitable simulators for tracheal pathology, have a learning value and recommend it to others for use in training.

**Conclusion:**

Using material jetting 3D printing to create patient-specific anatomical models is a promising modality of simulation training. Our results support further evaluation of the printed airway model as a bronchoscopic trainer, and suggest that pathological airways may be simulated using this technique.

## Background

Lung cancer is the leading cause of cancer death and accounts for a disproportionate burden of cancer-related mortality [1]. Advanced stages of lung cancer are associated with markedly poorer prognosis, with one-year survival at 72.5% for TNM stage I disease and 15.9% for stage IV disease [2].

Early diagnosis and staging by histological study are therefore essential in guiding clinical decisions and ultimately predicting disease progression. Suspicious lung nodules are usually benign but occasionally malignant; therefore, a clinical need exists for techniques that are minimally invasive, and at the same time are of sufficient diagnostic yield.

The approach to histological evaluation of a suspicious lung nodule depends primarily on its location. If proximal, a respiratory physician may obtain tissue endoscopically via the airways, using bronchoscopic biopsy of the lesion under ultrasonographic guidance. Peripheral nodules are preferentially sampled using computed tomography (CT) guided transthoracic needle biopsy by interventional radiologists [3].

Bronchoscopy is a versatile procedure for endoscopic exploration of the airways. Beyond biopsy of suspicious nodules, the bronchoscopic arsenal includes forceps, ultrasonographic probes, endobronchial tubes and even stent insertion, offering myriad diagnostic and therapeutic applications in the field of pulmonology [4].

Skilful bronchoscopy requires a working knowledge of bronchopulmonary segmental anatomy, as well as experience in endoscopic manoeuvres [5]. Being a key competency in respiratory physician training, numerous studies have evaluated acquisition of bronchoscopic proficiency by resident physicians [6, 7].

Procedural training in medical education must balance the importance of realistic training experience with risk of harm to patients by inexperienced operators. In this regard, simulation-based training with physical or virtual airway models eliminates patient risk and is recommended over traditional apprenticeship teaching [6–9], in which a trainee attempts the procedure on a patient under supervision by an experienced colleague [6–9]. Simulated modalities possess the advantage of re-enacting emergent situations in safe training environments; for experienced operators, it enables pre-procedural preparation for the difficult airway [10–12].

In developing these bronchoscopic simulators, important considerations are anatomical accuracy, fidelity (the degree of representation) to actual clinical scenarios, and cost [7]. To address these factors, bespoke three-dimensional (3D) printed models of the airway offer an advantage [13–18] over standardized task trainers, which are expensive and at best approximations of the bronchial tree, a complex segmented structure. Several groups have developed physical and virtual airway models varying in composition and complexity, including 3D printed models [17], papier-mâché [19], plastic phantoms [15], and virtual 3D reconstruction [20] as inexpensive and realistic alternatives to standardized task trainers.

Multi-material 3D printing, by means of customized colours and haptics, offers the potential for high-precision anatomical modelling which demands differentiation of tissue subtypes [21, 22]. The technique remains relatively unexplored in medical simulation, despite a growing body of research in anatomical education. Several groups have described applications in teaching complex segmental and branch anatomy [23], improvement in structure recognition [24], vis-à-vis cadaveric models [25], and 3D depiction of pathological entities to a postgraduate audience [26, 27]. A mixed-methods study in ASE supported 3D printing both as a standalone educational tool and as a complement to existing modalities of teaching. With regard to simulation, a limited number of previous studies have investigated this technique mainly for surgical simulation [28, 29], with promising results. There is a paucity of literature of its application in endoscopic simulation. To our knowledge, we have found no other study investigating multi-material 3D printed airway models. Our study aims to develop and evaluate the use of a novel multi-material 3D printed airway model of adult for both bronchoscopic anatomy teaching and flexible bronchoscopic simulation training.

## Methods

### Study Design

This study was granted institutional review board approval (IRB-2017-05-052). Following development of three patient-specific 3D printed airway models, this study evaluated its validity as an airway trainer by expert respiratory physicians, who tested the model and provided their opinion by means of a structured questionnaire.

### Development of Multi-material 3D Printed Models

Computed tomography (CT) scans of the thorax were obtained and anonymized from patients from the Diagnostic Radiology department in Tan Tock Seng Hospital (TTSH), Singapore with SOMATOM Definition AS for 256-slice series, SOMATOM Definition Flash for 128-slice series and Sensation Cardiac 64 for 64-slice series (Siemens Healthcare, Erlangen, Germany).

**Fig. 1:**
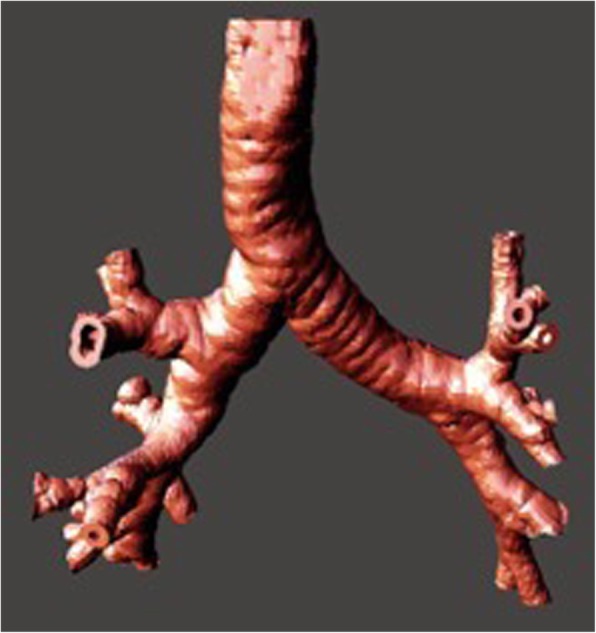
3D virtual model after reconstruction of the normal airways

A 256-slice series (1.00 mm slices) from a patient with normal lung findings, a 128-slice series (3.00 mm slices) from a patient with an obstructing right mainstem bronchus tumour, and a 64-slice series (3.00 mm slices) from a patient with tracheal compression by a retrosternal goitre were segmented using OsiriX Lite (Pixmeo SARL, Bernex, Switzerland) with a radiodensity-based threshold algorithm to isolate the radiolucent airways as a printable standard triangle language (.stl) mesh file. This initial model, representing the intraluminal space of the tracheobronchial tree, was then processed in Autodesk Meshmixer (Autodesk Inc., San Rafael, CA) where a surrounding airway wall of 2 mm could be fashioned, and the bronchi truncated at the third order of airways (Fig. 1). This uniform 2 mm layer substituted for airway wall structures including the trachealis and cartilage, which typically are undelineated with CT imaging.

Objet500 Connex3 (Stratasys Ltd., Eden Prairie, MN) was chosen as a 3D printer for its multi-material jet capable of fusing plastic and rubber [30]. The accuracy of material jetting 3D printing has been previously studied and concluded to be accurate [31, 32]. This technique was used to produce models of varying Shore hardness^1^ (the objective measure of material hardness by quantifying its resistance to indentation by a calibrated spring). Two different materials were used to achieve haptic representation: firstly, a custom mix of plastic (FullCure RGD851, VeroMagenta) and rubber (FullCure 930, TangoPlus) constituted the tracheal wall with Shore hardness values arbitrarily selected from the D40 – D60 range; and secondly, a fully rubber (FullCure FLX930, TangoPlus) material was used for the tumour. Support resin (FullCure, 705) was used for printing. Approximately 25 g of plastic and 50 g of rubber was used per model. Following this, manual post-processing with water and cleaning instruments was required to remove the partially water-soluble support resin from the final pieces.

### Study Participants

To validate the printed models as airway trainers we recruited expert respiratory physicians to perform bronchoscopy and provide feedback on their experience. Consultant respiratory physicians with at least six years’ formalized training in respiratory medicine, following an accredited residency programme based on standards set by the American Council for Graduate Medical Education (ACGME) working in association with Tan Tock Seng Hospital and the Lee Kong Chian School of Medicine were identified, and recruited to the study by email invitation. Prior to the study, informed consent was obtained and documented from participants.

### Conduct of Bronchoscopic Evaluation

**Fig. 2:**
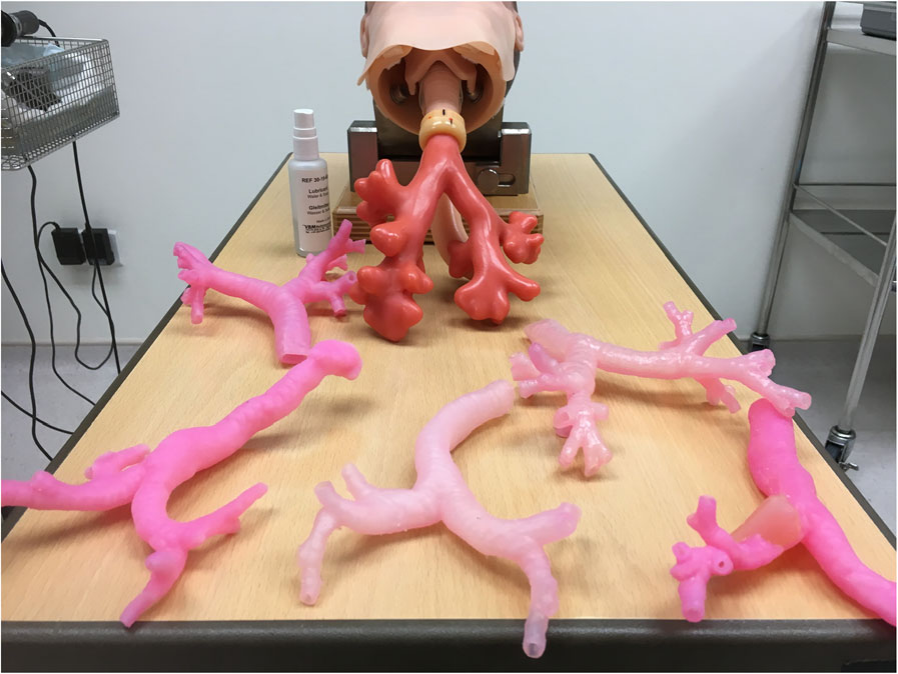
Standard airway trainer, comprising detachable lower airway model attached to upper airway structures. Surrounding this are a series of 3D printed models derived from CT scans as described in the Methods section

Before attempting bronchoscopy on the 3D printed models, all participants first performed flexible bronchoscopy using a standard endoscopic trainer Karl Storz 8402 ZX (KARL STORZ Endoskope, Tuttlingen, Germany) comprising a flexible bronchoscope coupled with a video monitor, with an open-mouth airway simulator model. This rubber-and-silicone airway trainer simulated both upper and lower airway anatomy, and incorporated a detachable tracheobronchial tree with an interface at the subglottic level (Fig. 2). The intention of this step was to allow the participants to familiarize themselves with the manoeuvring of the training bronchoscope, which differs from the model typically used in clinical practice.

Following familiarization with the bronchoscope, and participants performed the same procedure on the 3D printed airway models which were substituted for the original rubber tracheobronchial tree of the airway simulator, and were provided with a questionnaire to rate their experience vis-à-vis flexible bronchoscopy in their usual clinical practice.

### Design of Bronchoscopic Validation Metric

Taking reference from a related study on a paediatric laryngeal simulator made using a 3D printed mould [33], a questionnaire comprising ten five-point Likert scale items, ranging from 1 (strongly disagree) through 5 (strongly agree) and two open-ended items was developed (Table 1).

**Table 1:** Bronchoscopic evaluation questionnaire

Subjective rating items	
Anatomical Accuracy *Colour of the 3D printed model was similar to the endoscopic view of the real trachea*	
*Material used to print the trachea accurately reflected real tissue properties*	
*Endoscopic bronchial anatomy in the 3D printed model appeared similar to human anatomy*	
Clinical fidelity	
*Bronchial tree access was realistic on the 3D printed model 3D printed models accurately represented tracheal pathology*	
*Manipulation of the flexible bronchoscope was realistic in the 3D printed model*	
Perceived usefulness *I believe the 3D printed model is a useful tool for training basic bronchoscopic skills*	
*Given the opportunity I would like to use the 3D printed model again for training/practicing/demonstrating bronchoscopic skills*	
*I would recommend the 3D printed model as a tool for training/practicing/demonstrating bronchoscopic skills to colleagues/other trainees*	
*Training on the 3D printed model is a valuable learning exercise*	
Open-ended response	
*What is the strength of 3D printed airway models?*	
*How could the 3D printed airway models be improved?*	

### Statistical Analyses

Following the original paper^1^ [33] from which the validation tool was modified, reliability was calculated for all items as a composite. Rating responses were described, and open-ended responses were analysed for common themes.

## Results

### 3D printed models of airways

**Fig. 3:**
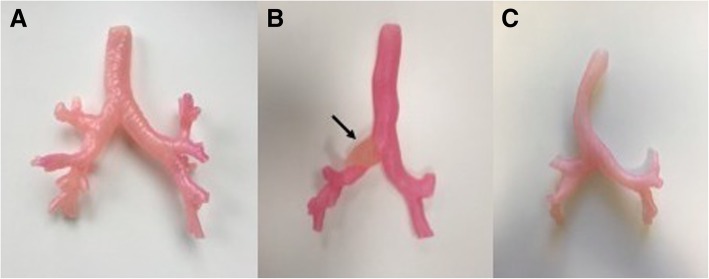
Three 3D printed models are shown: **a** Normal tracheobronchial anatomy to the extent of the third order of bronchi (segmental bronchi), **b** pathologic specimen, with occluding tumour (arrow), and **c** pathologic specimen, with displacement of the trachea by a retrosternal goitre

A total of three models were printed; these were patient-specific, life-sized and assigned both colour and specific Shore hardness. The proportion of plastic and rubber were adjusted to specific Shore hardness values; the resultant colour was dependent on the proportion of red plastic (FullCure RGD851, VeroMagenta) present in the print substrate. The first model of Shore hardness D50 (Fig. 3a) depicts the normal anatomy of airways from the trachea and its constituent branches including primary, secondary (lobar) and tertiary (segmental) bronchi. The second model of Shore hardness D60 (Fig. 3b) shows the proximal lobar bronchial divisions and a tumour obstructing the right mainstem bronchus. In the third model (Shore hardness D40, Fig. 3c) the trachea appears bowed because of extrinsic compression by a retrosternal goitre.

### Validation by Bronchoscopists

Five consultant respiratory physicians (four male, one female) responded to the invitation. All of them accepted the terms of participation and provided responses to the survey questionnaire. From the subjective rating responses, the calculated Cronbach’s alpha of 0.83 indicated reliability of the rating component of the questionnaire.

**Fig. 4:**
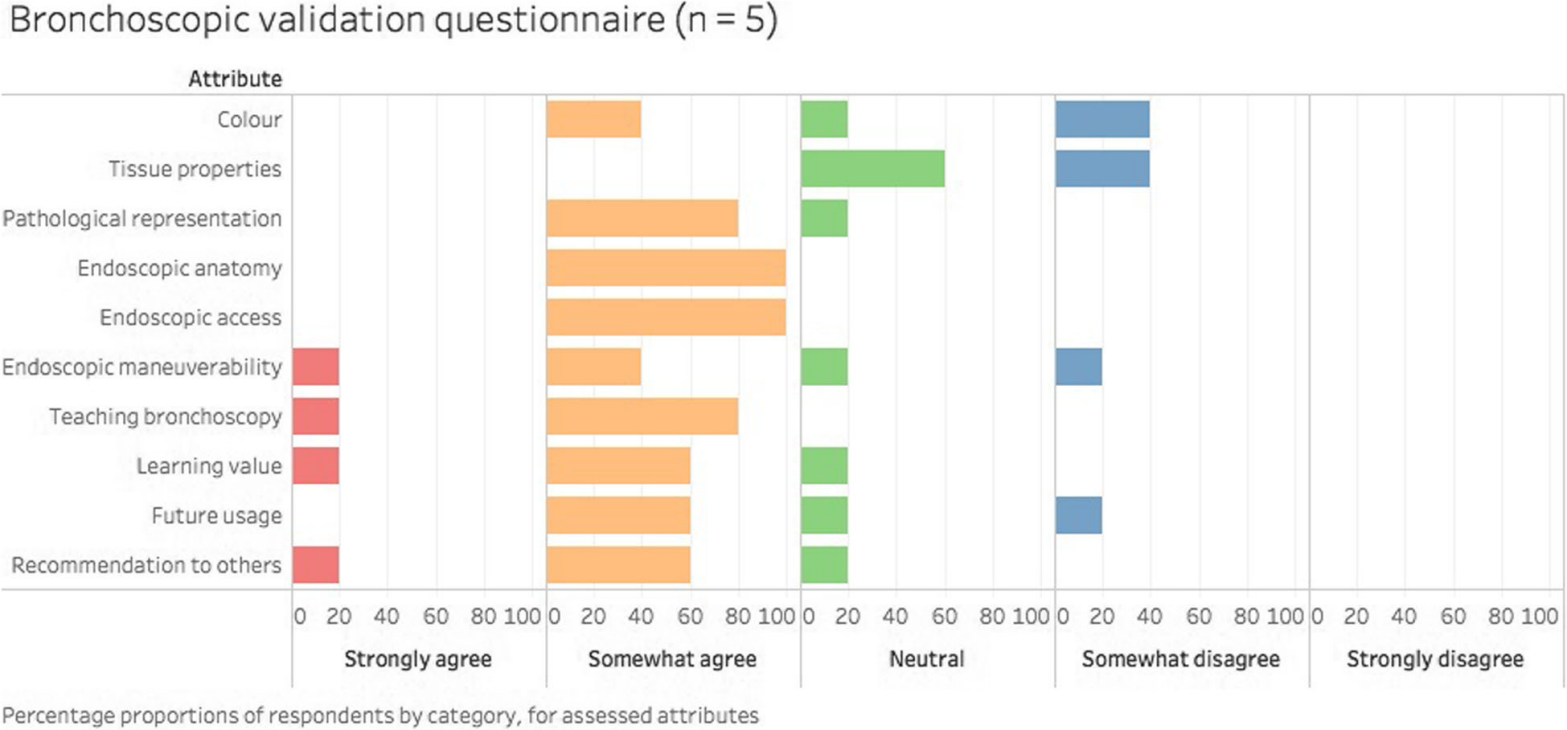
Bronchoscopic validation questionnaire responses in segmented bar chart

**Fig. 5:**
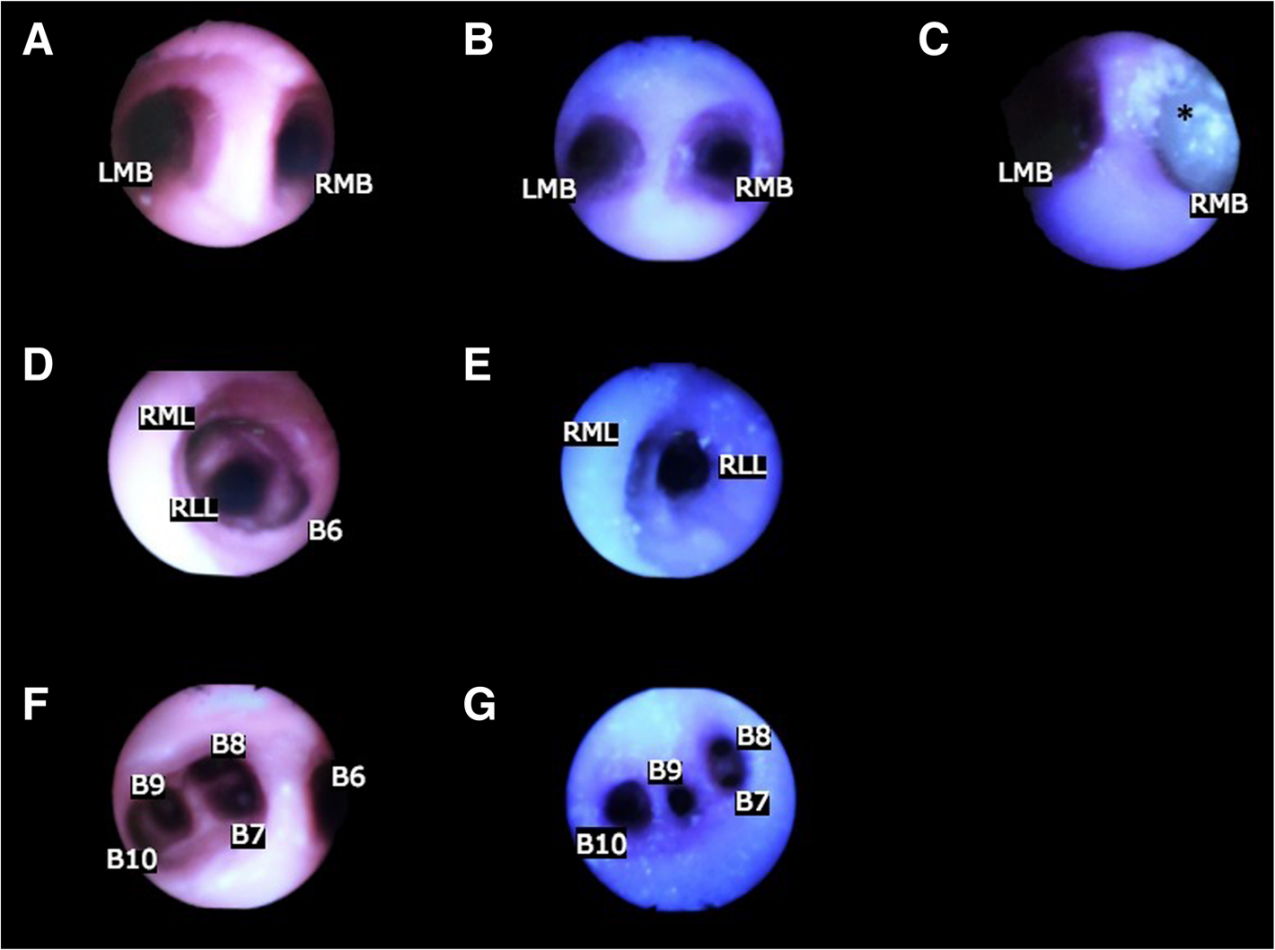
Upper row: bronchoscopic view of carina showing left mainstem bronchus orifice (LMB) and right mainstem bronchus orifice (RMB), as seen from **a** standard airway trainer, **b** 3D printed normal of normal anatomy and **c** 3D printed model of right mainstem bronchus tumour (*). Middle row: bronchoscopic view of right mainstem bronchus, as seen from **d** standard airway trainer and **e** 3D printed model of normal anatomy, showing right middle lobe orifice (RML), right lower lobe orifice (RLL) and superior basal segment orifice (B6). The tumour model is not shown because the model was truncated after the first order of bronchi. Lower row: Bronchoscopic view of left inferior lobar bronchus, as seen from **f** standard airway trainer and **g** 3D printed normal of normal anatomy showing superior basal segment orifice (B6), medial basal segment orifice (B7), anterior basal segment orifice (B8), lateral basal segment orifice (B9) and posterior basal segment orifice (B10)

The 3D printed model was generally well received by the bronchoscopists, with positive opinions of the anatomical accuracy (Fig. 4). All participants either agreed or strongly agreed that endoscopic anatomy and access are representative (Fig. 4, Fig. 5) – citing “the anatomy of the airways are realistic”. Majority of the respondents felt that manipulation of the bronchoscope and representation of tracheal pathology was realistic in the model, and supported its use as a trainer – citing “less expensive” cost, the ability to “provide greater spectrum of pathologies” and “provide for EBUS-TBNA [endobronchial ultrasound-guided transbronchial needle aspirate] training”. However, all participants were neutral or disagreed with the realism of the tissue properties, commenting that “the texture is somewhat rough/harder than normal; the colour is not quite the same”. Several requests were made to improve the model in the open-ended responses, including modelling of upper airways - “adding the vocal cord for intubation purposes”, removal of residual supporting material from the interior of the model, and replicating more realistic colour and texture. Nonetheless, the majority of the participants felt that 3D models created learning value and would recommend to others for training.

## Discussion

In this study, we developed 3D printed airways by material jetting technology and validated their efficacy as a bronchoscopic trainer. To our knowledge, this is the first multi-material print of the tracheobronchial tree. Other investigators have developed models with reasonable realism, but that require additional post-print processing, such as painting [34], or silicone coating and mould-casting of a polyp [17]. In this study, we also used multi-material printing to demonstrate the single-stage synthesis of a flexible coloured model with embedded tumour. This conserves the lead time in production and ensures standardized fabrication across print runs, which are important factors influencing the clinical application of these models.

Furthermore, the capability of multi-material printing to admix two or more materials allows one to exploit desirable attributes of both materials; in this case, the strength of plastic and the flexibility of rubber in a custom plastic-rubber blend are superior to either in isolation as a choice for the luminal material. Single-material prints of flexible rubber are prone to damage, requiring support to prevent from bending during the bronchoscopy procedure [17], while single-material prints of rigid plastic do not offer the pliability of the plastic-rubber blend. However, it must be noted that the hardness of the material being the primary variable, as was in this case, inevitably affected the secondary variable of colour. Colour was also determined by the proportion of the constituent materials, with the hardest model (Fig. 3b) deeper in colour, and the softest model (Fig. 3c) a lighter hue of pink. Our validation questionnaire affirmed high clinical fidelity of the model, but received ambivalent appraisal regarding the colour and texture of the internal wall. We envisage refinement of these factors in subsequent print runs with the multi-material technique. In addition, future research is needed to evaluate student perceptions and the effectiveness of 3D printed airways in achieving learning outcomes.

It is important to note that where endoscopic simulators are concerned, the required physical characteristics differ somewhat from a surgical incision-site simulator. Being an intraluminal procedure with manoeuvres performed in “negative” space, more emphasis is placed on anatomical accuracy and user navigational experience than the consistency of the wall material. Our study questionnaire found that the anatomical accuracy of the 3D printed model was well-received by expert bronchoscopists. Our model, made at a cost of SGD~$135, offers an inexpensive modality for depicting to-scale real anatomy and a wide range of pathologies. Owing to the versatility of 3D printing, it is conceivable to design a series of anatomical and pathological models (patient specific) that may be affixed to a single commercial task trainer, sparing the cost of simulating a range of clinical scenarios. The prime advantage of 3D printed simulators is that of patient-specific modelling, which can conceptualize unique clinical situations. For instance, where variant anatomy or pathological distortion of the airways is encountered, 3D printed airway models accurately represent pathoanatomy [11, 33, 35], enabling pre-procedural planning of the endoscopic approach. Preparedness for the difficult airway improves outcomes [10, 12] in a patient population at higher risk of technical complications related to prolonged sedation, injury from instrumentation, and procedural failure necessitating repeated investigation. Considerations of time and cost currently limit the use of 3D printed airway modelling to case series with complex anatomy [36], but we anticipate that improvements in efficiency will see its broadened clinical use for pre-procedural preparation and optimization of surgical outcomes.

### Limitations

Only five expert bronchoscopists participated in our study. For comprehensive validation of the model, a larger pool of both expert and trainee bronchoscopists is required, ideally in a blinded study comparing standard bronchoscopic trainers to the 3D printed airway model.

## Conclusions

Three multi-material 3D models of the airway were developed and validated by expert bronchoscopists for simulation training of flexible bronchoscopy. One normal and two pathological patient-specific, life-sized airway models were made to represent normal anatomy, an anatomical lesion, and extrinsic tracheal compression. With minor refinements, 3D printed models shows potential for patient-specific bronchoscopic training, particularly in the setting of airway pathology.

## Data Availability

Data analyzed during this study are included in this published article.

## Abbreviations

2D: Two-dimensional
3D: Three-dimensional
ACGME: American Council for Graduate Medical Education
ASE: Anatomical Sciences Education
CT: Computer Tomography
EBUS-TBNA: [endobronchial ultrasound-guided transbronchial needle aspirate] training
SGD: Singapore Dollar
STL: Stereolithography

## Footnotes

1 Shore scale measures the hardness of a material by testing the resistance of the material against a device called the durometer. The durometer is an spring-loaded indenter that applies a force to the material. Should the material be easily indented, it will have a lower Shore hardness. If there is no penetration of the material at all, the material is said to have maximum Shore hardness (100). Two different indenters are used depending on the material composition: Shore A (loading force of 822 g) for soft elastomers and polymers, and Shore D (loading force of 4536 g) for hard elastomers and polymers.
